# Improving Valvular Pathologies and Ventricular Dysfunction Diagnostic Efficiency Using Combined Auscultation and Electrocardiography Data: A Multimodal AI Approach

**DOI:** 10.3390/s23249834

**Published:** 2023-12-14

**Authors:** Takeru Shiraga, Hisaki Makimoto, Benita Kohlmann, Christofori-Eleni Magnisali, Yoshie Imai, Yusuke Itani, Asuka Makimoto, Fabian Schölzel, Alexandru Bejinariu, Malte Kelm, Obaida Rana

**Affiliations:** 1 Mitsubishi Electric Inc., Kamakura 247-0056, Japan; 2 Division of Cardiology, Pulmonology and Vascular Medicine, Medical Faculty, Heinrich-Heine-University Düsseldorf, 40225 Düsseldorf, Germany; benita.kohlmann@uni-duesseldorf.de (B.K.); obaida.rana@med.uni-duesseldorf.de (O.R.); 3 Data Science Center/Cardiovascular Center, Jichi Medical University, Shimotsuke-City 329-0498, Japan; 4 CARID—Cardiovascular Research Institute Düsseldorf, 40225 Düsseldorf, Germany

**Keywords:** auscultation, electrocardiography, valvular disease, heart failure, multimodal artificial intelligence

## Abstract

Simple sensor-based procedures, including auscultation and electrocardiography (ECG), can facilitate early diagnosis of valvular diseases, resulting in timely treatment. This study assessed the impact of combining these sensor-based procedures with machine learning on diagnosing valvular abnormalities and ventricular dysfunction. Data from auscultation at three distinct locations and 12-lead ECGs were collected from 1052 patients undergoing echocardiography. An independent cohort of 103 patients was used for clinical validation. These patients were screened for severe aortic stenosis (AS), severe mitral regurgitation (MR), and left ventricular dysfunction (LVD) with ejection fractions ≤ 40%. Optimal neural networks were identified by a fourfold cross-validation training process using heart sounds and various ECG leads, and their outputs were combined using a stacking technique. This composite sensor model had high diagnostic efficiency (area under the receiver operating characteristic curve (AUC) values: AS, 0.93; MR, 0.80; LVD, 0.75). Notably, the contribution of individual sensors to disease detection was found to be disease-specific, underscoring the synergistic potential of the sensor fusion approach. Thus, machine learning models that integrate auscultation and ECG can efficiently detect conditions typically diagnosed via imaging. Moreover, this study highlights the potential of multimodal artificial intelligence applications.

## 1. Introduction

The development of cardiac diagnostic imaging tools increased the reliance on these methods for the diagnosis and treatment of cardiovascular diseases [1,2]. Consequently, the use of traditional diagnostic techniques, including auscultation and electrocardiography, is declining. However, the number of hospitals with diagnostic imaging facilities is limited, thus restricting easy access for patients [3]. Enhancing the diagnosing accuracy of accessible traditional testing methods, which are accessible even in small-scale clinics, can promote early diagnosis and treatment. This may not only alleviate the burden on patients but also reduce economic strain [4].

Recent research has increasingly highlighted the efficacy of artificial intelligence (AI) in diagnostic processes utilizing simple testing methods [5,6,7]. Nevertheless, these studies typically focus on singular modalities. This contrasts with real-world clinical scenarios, where physicians integrate data from a variety of tests for patient evaluation and diagnosis. Further, recent developments suggest that the utilization of a stacking approach involving the amalgamation of multiple machine learning models enhances the performance of predictive models [8].

Based on this, we hypothesized that combining various simple diagnostic approaches might significantly improve the accuracy of AI-driven diagnostics.

These simple diagnostic sensors should be easily adoptable in daily clinical practice. Therefore, 12-lead electrocardiography (ECG) and auscultation were used as sensors. In this study, we aimed to create an AI model using data from 12-lead ECGs and auscultation to detect severe valvular disease (severe aortic valve stenosis (sAS) and severe mitral valve regurgitation (sMR)) and left ventricular dysfunction (LVD) with high accuracy. Furthermore, we compared the contribution of both techniques in the diagnosis of each of the conditions.

## 2. Materials and Methods

### 2.1. Research Participants and Data Collection

In this study, we extracted data from a previously reported database [9]. The inclusion criteria were (1) age ≥ 18 years, (2) availability of a complete set of echocardiography tests, (3) availability of printed 12-lead ECG recordings on the same day of echocardiography, and (4) willingness to provide written informed consent. The exclusion criteria were (1) a history of heart valve surgery or transcatheter aortic valve implantation and (2) use of a cardiac implantable electronic device, except an implantable loop recorder. The diagnosis was based on existing guidelines for echocardiography [10,11]. None of the patients were enrolled twice in the present study.

The auscultators were blinded to the patients’ clinical information at the time of recording. A phonocardiogram (PCG) was recorded using an Eko Duo system (Eko Devices Inc., Oakland, CA, USA) on the day of echocardiography (±1 d). The patients’ heart sounds were recorded at 4000 Hz (.wav format) at three auscultation locations, including the second intercostal space along the right sternal border (L1: 2RSB), Erb’s area (third intercostal space along the left sternal border, L2: ERB), and apex (fifth intercostal space in the midclavicular line: L3: APX), for 15 s.

Furthermore, a 12-lead ECG was conducted on the same day. The ECG was printed and scanned in PNG format (12 lead, limb lead, and precordial reads; resolution, 3187 × 1840 pixels). Clinical data, including medical and treatment history, were collected on the day of the PCG recording.

Overall, 1052 individuals who underwent ECG were enrolled in this study (Table 1 and Table 2). Moreover, to enroll an equal number of participants with target cardiovascular pathologies, participants with sAS, sMR, and severe left ventricular dysfunction (sLVD) were preferentially assigned as previously documented [9].

All data were collected after written informed consent was obtained from the patients, and the participants’ data were pseudonymized at the data center. Furthermore, the data were anonymized during the analyses in this project.

### 2.2. Dataset Preparation

A modified stratified 4-fold cross-validation method was adopted to train the model. We organized the established dataset according to the severity of AS, MR, and LVD to balance the number of AS, MR, and LVD cases in the training, development, and test datasets and to build four independent training/development data groups without overlap. The test cases were common to all four groups.

Details of the training/development dataset (cross-validation set) are presented in Table 1, and details of the test dataset are presented in Table 2.

### 2.3. Data Preprocessing

#### 2.3.1. PCG

**Figure 1:**
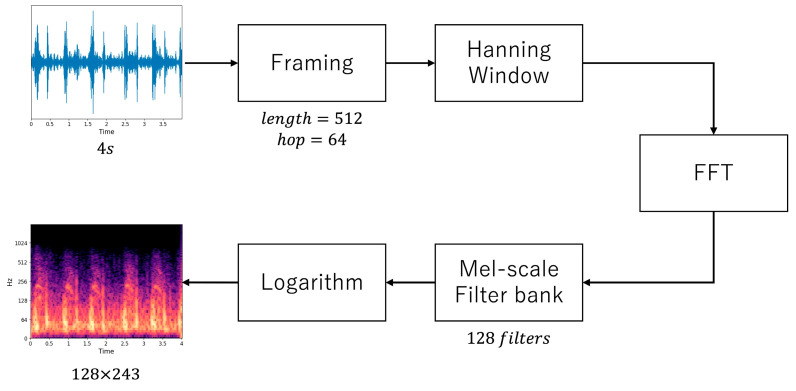
Preprocessing steps of heart sound data. Signal preprocessing for phonocardiogram is presented. FFT = fast Fourier transform.

In this study, the heart sound data were not standardized or normalized, as these procedures did not affect the efficiency of model training during the preliminary experiments. For preprocessing, 128-dimension log-Mel spectrograms were extracted based on the sound (.wav format) data using the following steps (Figure 1):

Oversampling: Each record was divided into multiple data of 4 s based on predefined time steps depending on the severity of each target disease (Table 3).

Fourier transform: Short-time Fourier transform was computed using the Hann window, 512 sample windows, and 64 samples of hop size.

Mel-scaled filter bank: We applied the Mel-scaled filter bank (128 filters) followed by the logarithm.

We used SpecAugment (frequency mask and time mask) and mix-up for data augmentation only during training. The data shape after preprocessing was 243 × 128 (the input shape of the convolutional neural networks (CNN)).

#### 2.3.2. ECG

The ECG scans were cropped for disease detection to extract relevant regions from the original scan to improve the accuracy and efficiency of disease detection. Three cropping techniques were used to focus on specific areas of interest.

Cropping for 12 leads:

A square region (size, 1840 × 1840 pixels) was extracted from the original ECG image (3187 × 1840 pixels). This region was selected to extract the complete waveform information across the 12 leads simultaneously. This approach provides information on the overall cardiac activity and detects anomalies that may appear across different leads.

Cropping for limb lead and precordial lead:

A square region of size 960 × 960 pixels was extracted from either the upper or lower half of the original ECG image to analyze specific regions. The upper half represents the limb-lead region, while the lower half represents the precordial-lead region.

Resize:

After extracting the relevant regions using the cropping process, the images were resized for standardization and ease of further processing. The 12-lead images were resized to 512 × 512 pixels, while the limb- and precordial-lead images were resized to 256 × 256 pixels. This resizing ensures a balance between preserving important details and reducing computational complexity.

Horizontal shifting augmentation:

The horizontal shifting process involves repeatedly cropping and resizing the original ECG image as described above while shifting the cropping window horizontally by a predetermined offset during each iteration. The number of images extracted from an original image depended on the severity of the target disease (Table 3).

### 2.4. Training and Development of Models including Stacking

#### 2.4.1. Architecture of Model

**Figure 2:**
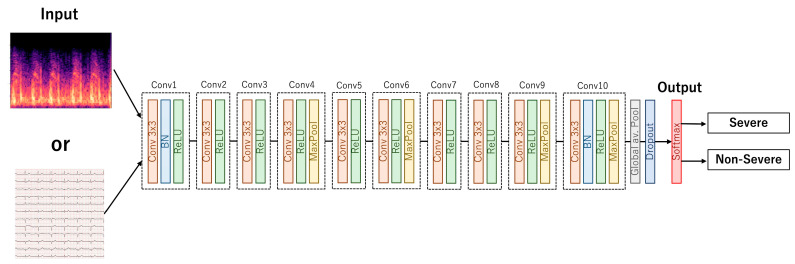
Architecture of convolutional neural network.

We used a 10-layer CNN based on our previous publications (Figure 2) [9,12]. The output of our models indicated whether the input heart sound or ECG signal indicated severe disease. The models were separately trained for each disease (AS, MR, and LVD). Python 3.7.6 and PyTorch 1.4.0 were used for this project. The equipment and the training conditions are provided in Supplementary Table S1.

Our model had 10 convolutional layers, followed by a ReLU activation function combined with batch normalization in the first and last convolutional blocks, based on our previous work. The 4th, 6th, 9th, and 10th convolutional blocks have max-pooling layers (MaxPool). Following the 10th convolutional block, a global pooling layer was set, and the final layer was activated using a softmax function.

We trained the CNN models using the 4 s heart sound data from all three collection locations and using the preprocessed ECG image data from all three patterns (12 lead, limb lead, and precordial lead), and then separately for each heart sound location and each ECG pattern. Six models were trained per training/development data group; thus, twenty-four models were trained in four groups.

#### 2.4.2. Model Stacking

**Figure 3:**
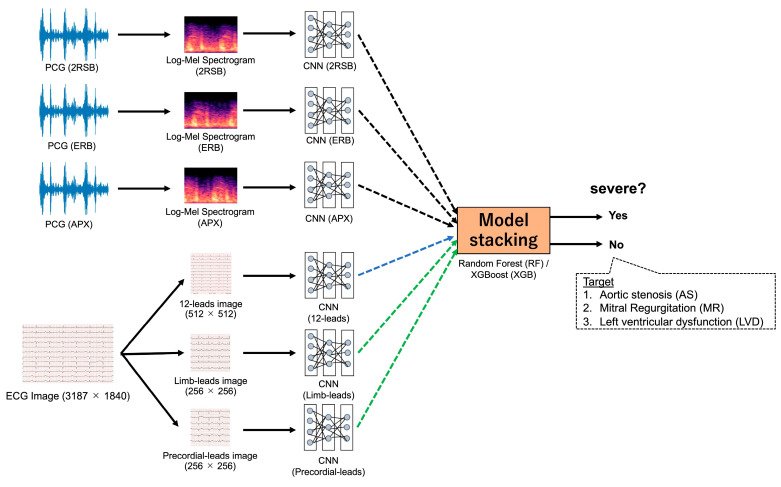
Sequential flow from input data to stacking. The sequential flow from input data to stacking is shown. See texts for details.

We used stacking CNNs for both the ECG and heart sound modalities. Figure 3 presents the sequential flow from input data to stacking. Model stacking was performed using two types of models: Random Forest (RF) and XGBoost (XGB).

The stacking patterns for the models were defined based on the four patterns presented in Table 4: Each stacking pattern was constructed separately for each training/development group. The goal was to leverage the complementary information extracted from the ECG and auscultation. During stacking, anomaly probabilities from individual CNNs were used as intermediate inputs and served as features to train the stacking models. By combining the predictions from the stacked models, we aimed to improve the disease detection capabilities.

The stacking model was trained using the development dataset. Hyperparameter tuning was performed using a grid search to maximize the F1 score. The stacking process using Random Forest and XGBoost was performed using scikit-learn 0.23.2 and xgboost 1.6.2.

#### 2.4.3. Model Selection

For model selection, we evaluated all available models, including individual CNNs for heart sound and ECG data, as well as the stacking models. The evaluation was performed using test data. The area under the curve (AUC) of the receiver operating characteristic (ROC) curve was used to assess the performance of the models, considering their ability to accurately detect positive cases.

As detailed above, we trained 14 distinct models, comprising both single and stacking models, across each cross-validation fold. The model with the highest AUC for the test data was subsequently selected from the models of each method within the four cross-validation folds. To statistically compare the performances of these models, we used the bootstrapping method, sampling with replacement 2000 times. We then used analysis of variance (ANOVA) for these bootstrapped metrics to discern differences among the models. Subsequently, we performed post hoc comparisons using the Tukey–Kramer honestly significant difference (HSD) test to identify specific group discrepancies. Based on this analysis, the top three models that emerged as the most promising candidates for disease detection were identified.

#### 2.4.4. Clinical Validation

We prospectively enrolled 103 patients as the clinical validation cohort to assess the performance of the models and the diagnostic contribution of each dataset (Table 2). These cases did not overlap with the individuals in the establishment cases.

#### 2.4.5. Modal Contribution Analysis

In this study, we aimed to estimate the significance of each modality incorporated into the stacking process via modal contribution analysis. We quantified these contributions using both the scikit-learn and XGBoost libraries. To bolster the reliability of our findings, we trained an identical model with fluctuating random seed values on 50 occasions for each training/development set (i.e., within the same cross-validation cohort). Subsequently, we computed statistical metrics, including the mean and standard deviation, for the modal contributions sourced from all models within each training/development set. Through this rigorous analysis, we gained insights into the relative significance of each modality within the stacking model and its respective roles in disease detection.

### 2.5. Statistical Analysis

Continuous data were presented as mean ± standard deviation for normally distributed data. Categorical data are presented as numbers and percentages. Non-normally distributed data were presented as median values (lower–upper quartiles). The chi-square test, Kruskal–Wallis test, Student’s *t*-test, or Fisher’s exact test were performed when appropriate. For the global test statistics, we used a significance level of 5%. Analyses were performed using the JMP software version 14 (SAS Institute, Cary, NC, USA) and custom Python scripts on MacOS computers.

## 3. Results

### 3.1. Performance of Single-Modal AI

**Figure 4:**
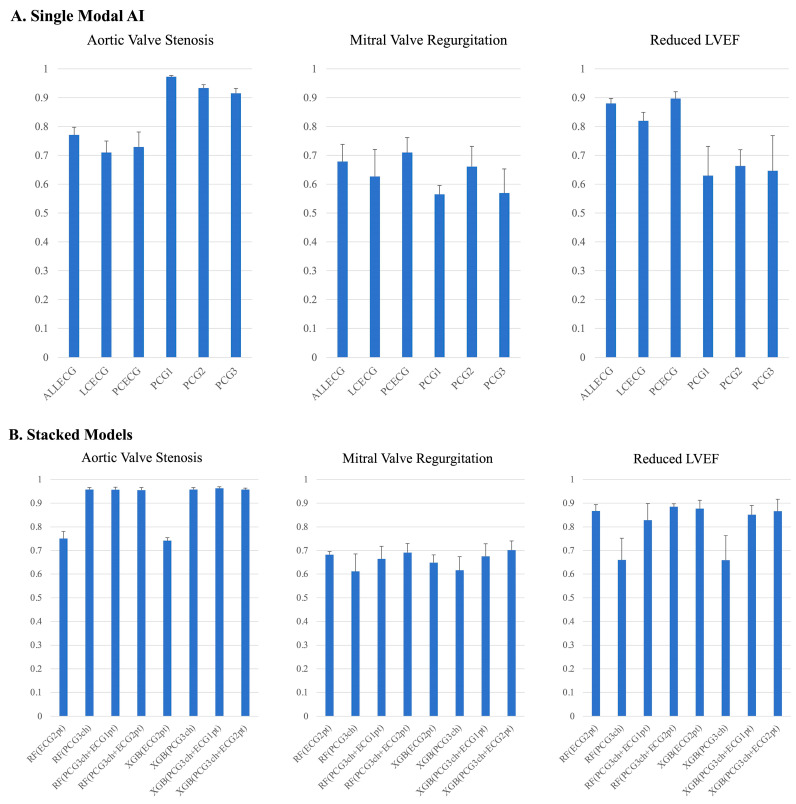
(**A**) Performance of trained AI for disease detection in the test cohort. (**A**) The performances (area under the receiver–operator characteristics curve (AUC)) of single-modal AI using a test cohort are presented. The AUC values varied depending on the pathologies. For severe AS detection, the models using phonocardiograms performed significantly better as compared to those using ECGs. For severe MR detection, there were no statistically significant differences in performance of the single-modal models. For severe LVD detection, the performance of PCECG and ALLECG models was better as compared to PCG1 and PCG3 models. ALLECG = 12-lead ECG; LCECG = limb-lead ECG; PCECG = precordial ECG; PCG1 = phonocardiogram from 2nd intercostal right sternum border; PCG2 = phonocardiogram from Erb; PCG3 = phonocardiogram from apex; RF = Random Forest; XGB = XGBoost; ECG1pt = 12-lead ECG in one sheet; ECG2pt = limb-lead and precordial-lead ECG separately. (**B**) Performance of trained AI for disease detection in the test cohort. (**B**) The performances (AUC) of stacked AI using a test cohort are presented. For severe AS detection, the models utilizing at least one phonocardiogram data performed significantly better than the models using only ECG data. For severe MR detection, there were also no significant differences in the performance of models. In contrast to the results of AS detection, for severe LVD detection, the models utilizing at least one ECG data point performed significantly better than the models using only phonocardiogram data. For abbreviations, see Figure 4A.

Figure 4A presents the results of each single-modal AI in the test dataset to detect sAS, sMR, and rEF, trained using a 12-lead ECG (ALLECG), limb-lead ECG (LCECG), precordial-lead ECG (PCECG), and heart sounds from three locations.

In the AS model, the model trained with heart sounds from 2RSB performed the best (AUC = 0.97), followed by the models trained with heart sounds from Erb (AUC = 0.94) and apex (AUC = 0.91). The performance of the models trained with ECGs was significantly inferior to that of the heart sound models.

For the MR model, although the performance disparity between the models was smaller than that of the AS models, the models trained with ECGs, particularly from the precordial-lead ECG, tended to perform better than the other models, followed by the models trained with the Erb heart sounds.

In the rEF model, the performance of models trained with ECGs, particularly the precordial-lead ECG and 12-lead ECG, was superior to that of the models trained with heart sound models.

### 3.2. Stacking and Diagnostic Performance

We combined the trained models using single-modal data using the proposed stacking techniques and tested their performance in detecting the three cardiac pathologies. Based on the results from the single-model AI, we used 12-lead ECG information in all cases. To evaluate the contribution of the limb and precordial leads, we tested the performance of the 12-lead ECG as a single diagram or as separately stacked limb and precordial leads. Figure 4B presents the performance of the stacked models and a comparison of the models. These results suggested that the optimal sensor may differ based on the target pathology. However, determining the superior model among the various models was not conclusively possible due to the lack of statistical significance.

Therefore, bootstrapping was performed to statistically evaluate the performance of each model for the three pathologies, as presented in Table 5, Table 6, Table 7.

In the AS model, the model with the highest AUC was trained with all three heart sounds, and 12-lead ECGs were stacked using XGB. The model trained only with heart sounds from 2RSB and the model with the three heart sounds and the limb-lead and precordial electrocardiograms separately stacked with RF had a good performance.

In the MR model, the model with the best performance was the standalone model with a 6-lead precordial ECG, followed by the model trained with all three heart sounds and electrocardiograms separately stacked by the limb and precordial leads using XGB, followed by the model using RF.

For the rEF model, the model stacking the limb- and precordial-lead ECG separately by XGB performed the best, followed by the model trained with the three heart sounds and the limb- and precordial-lead ECG tacked separately using XGB and the standalone model trained with the 12-lead ECGs as a single diagram.

### 3.3. Performance in Clinical Validation

**Figure 5:**
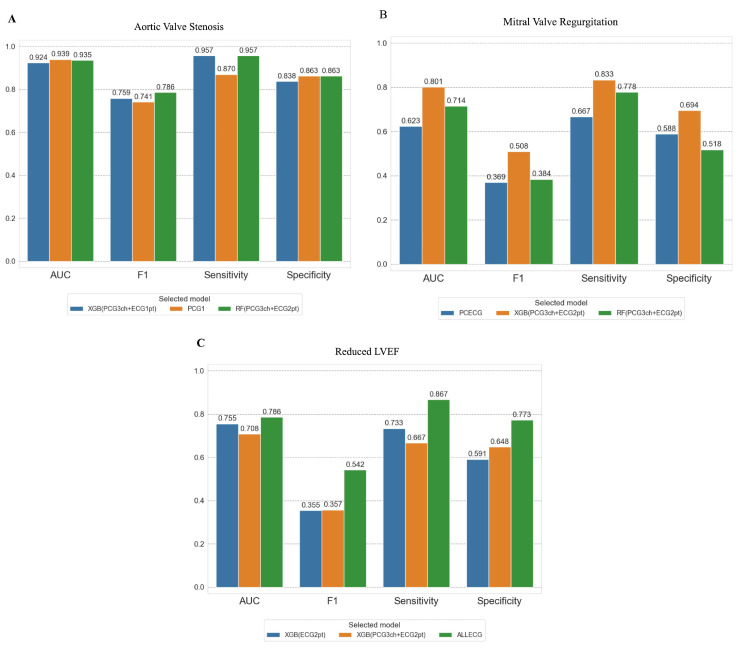
Performance of selected models in the clinical validation cohort. The top three performance models are presented for each pathology. The performance of the models did not vary for aortic valve stenosis detection (**A**). The performances of the models differed in the detection of mitral valve regurgitation (**B**) and reduced LVEF (**C**).

We tested the top three models selected for the clinical validation cohort (Figure 5). The performance differences observed for detecting severe AS were small among all models. However, performance variations were observed in the clinical validation of the chosen three models for MR and rEF. Compared to the test data, the performance in the clinical validation cohort was generally inferior, and the decline in performance was particularly pronounced in the rEF detection models.

### 3.4. Contribution of Each Modal to Detection

**Figure 6:**
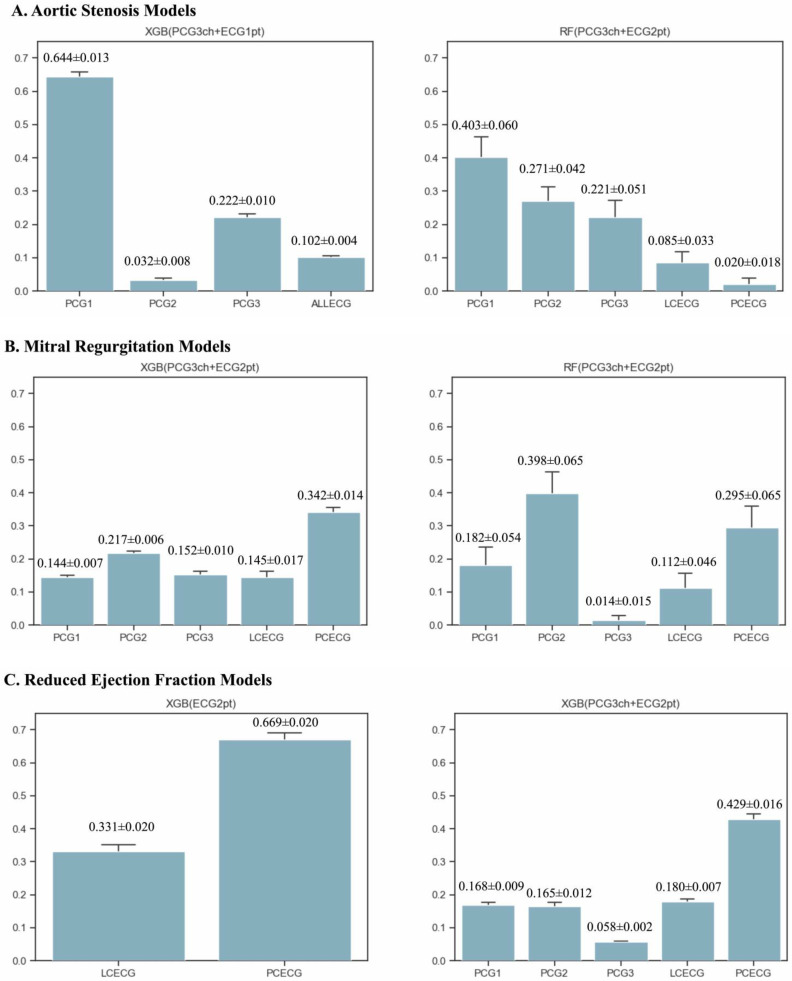
Contribution of each modal for disease detection. The contributions of each data point in detection of aortic valve stenosis (**A**), mitral regurgitation (**B**), and reduced LVEF (**C**) are presented. Data with high contributions differed among the pathologies. For the detection of aortic valve stenosis, all factors included in the stacking models showed significantly different contributions (*p* < 0.0001), and the phonocardiograms contributed more in general. For the detection of mitral regurgitation and reduced LVEF, the contribution of the precordial-lead ECG was higher than that of the limb-lead ECG. PCECG and PCG2 contributed significantly more in the detection of severe MR (*p* < 0.0001). PCECG and LCECG played a larger role in the detection of reduced LVEF. Abbreviations are the same as in Figure 4.

Figure 6 illustrates the contribution of each modality to the selected models, calculated after 50 training cycles in each cross-validation cohort. For AS detection in model 1, the contribution of the 2RSB heart sound data was the highest, followed by the apex and Erb heart sounds. The contribution of the 12-lead ECG was significantly lower than that of the heart sounds. Model 2 was a standalone 2RSB heart sound model. In model 3, the contributions were in the order of 2RSB > Erb > apex heart sounds. Although limb- and precordial-lead ECGs were incorporated into model 3, their contributions were significantly lower than those of heart sounds for the detection of severe AS.

For MR detection, model 1 was selected as the standalone precordial-lead ECG model. In model 2, the contribution of the precordial-lead ECG was the highest, followed by that of the limb-lead ECG, which was significantly higher than that of the heart sound. In model 3, the contribution of the precordial-lead ECG was the highest, followed by the 2RSB heart sound and limb-lead ECG. Moreover, the contribution of the apex heart sounds was lower than that of the other information.

In the case of rEF in model 1, the precordial-lead ECG contributed significantly more than the limb-lead ECG. In model 2, the contribution of ECG in detection, particularly by the precordial lead, was higher than that of heart sounds. Model 3 incorporated the 12-lead ECG as a single diagram.

## 4. Discussion

This study revealed that efficient detection of AS, MR, and LVD is possible using the appropriate combination of multiple sensors, i.e., multiple points of auscultation and ECG. This multi-sensor strategy not only offers a novel method but also may enhance model performance in the medical field.

Moreover, this study demonstrated that the contribution of auscultation and ECG data to the detection of each disease varies. These results suggest that when considering detection models for these diseases, a “one-size-fits-all” approach is inadequate, and modeling should be tailored to the target disease. The integration of information from multiple sensors, including simple and traditional diagnostic tests using AI, can result in increased diagnostic capabilities. The tailored modeling approach incorporating simple clinical tests may enhance clinical decision-making processes.

Auscultation was significantly more important than ECG for detecting AS. This was corroborated by clinical validation, with no difference in accuracy between model 1, trained with ECG data, and model 2, trained using only auscultation data. In a previous study, we reported favorable screening capability of heart sound classification AI in identifying severe AS [1]. The achieved AUC was 0.93, which was comparable to the models in the present study. Furthermore, Cohen-Shelly et al. reported the use of ECG-AI in detecting AS with asymptomatic severe AS efficiently screened to avoid sudden cardiac death [13]. Their ECG model achieved an AUC of 0.85, which was comparable to our models using only ECG data. The stacked models in the present study combining ECG and PCG data demonstrated an AUC of 0.93, while the model combining ECG and age/sex by Cohen-Shelly showed an AUC of 0.87. Future research is needed to determine if adding a variety of data improves the models.

Data from precordial-lead ECG are crucial for MR diagnosis. The stacking model that combined the 12-lead ECG (integrating both limb leads and precordial leads) with auscultation data demonstrated marginally improved detection accuracy in the clinical validation group as compared to the model trained only with precordial leads. A previous study by Chorba et al. demonstrated that the deep learning model could detect severe MR efficiently (AUC 0.86), though they built the models using the auscultation data from severe MR and no MR [14]. Elias et al. reported higher MR detection (AUC = 0.83) based solely on ECG [15]. Their use of approximately ten times more ECG data may account for the enhanced performance of their models.

For the detection of LVD, the contribution of auscultation data was much lower than that of ECG. The performance of model 2 suggests that the addition of the auscultation data to the ECG data did not improve disease detection in the clinical validation cohort. Attia et al. reported that even a single-lead ECG could detect a low ejection fraction (AUC = 0.88) [16]. The study by Bachtiger et al. also showed that the ECG screening could detect reduced LVEF (AUC = 0.85) [17]. In their studies, a digital stethoscope recording a single-lead ECG was used, which may enable the use of a combination of auscultation and ECG data in clinical practice. In the present study, the clinical validation cohort had a higher percentage of severe LVD as compared to training/development/test cases and also as compared to the report by Attia and Bachtiger [16,17]. This difference in patients’ characteristics might have affected the results.

In each case, two of the top three models were stacked models, integrating multiple modalities. This indicates that the integration of different modalities through stacking may improve diagnosis efficiency. Thus, the ability to automatically adjust the weight of each modality and use the appropriate modality depending on the disease may enable high-accuracy detection, even when the target disease changes.

Moreover, by using stacking models, the effect of each modality’s output on disease detection becomes evident, enabling assessment of the relative importance of different modalities. Thus, the interpretability of machine learning models can be improved, and decisions on modality selection and combination decisions may become more evidence-based [18]. This approach is also effective for gaining insights into each modality and target disease and may contribute to the development of more effective disease diagnostic methods. In this study, for MR and LVD detection, the precordial leads played a greater role than the limb leads. To the best of our knowledge, this is the first study to validate this in a clinical setting. Further research is needed to identify waveforms of precordial lead that have diagnostic value. Even in the modern age, with advancements in imaging diagnosis, screening with high accuracy using simple tools such as ECG and auscultation can alleviate the economic burden on the patients. This study suggests that evaluating the contribution of data to AI-based diagnosis can help reevaluate the value of traditional medical information.

Furthermore, one of the top three models in each case used only a single modality. Thus, high-accuracy detection may be attained even with a single modality, provided that the appropriate modality is selected. This was supported by the clinical validation for LVD, where the model using only a 12-lead ECG, combining the limb- and precordial leads in a single diagram, yielded the best accuracy.

We explored three common cardiac pathologies in the present study. Future research should focus on enhancing the precision of models that integrate simple clinical tests, such as blood pressure and oxygen saturation measurements. These tests could become a routine part of clinical practice, especially in non-specialized healthcare settings. We anticipate that validating AI models using these simple clinical tests will revolutionize clinical routines in the age of digital transformation.

This study had some limitations. This was a single-center study with no external validation. However, in the clinical validation group, the time of data collection was separated from that of the construction group with no duplication of patients; therefore, no data leakage was expected. From an informatics perspective, the performance of the original model is crucial, as the effectiveness of stacking relies on the underlying neural network’s ability to make accurate inferences. If the original neural network is unable to perform well, stacking may not yield significant improvements. Additionally, depending on the specific disease being targeted, it may be necessary to modify the model architecture, including the incorporation of non-DNN models. Furthermore, there is a possibility of improving accuracy by combining various model types in addition to multimodal stacking. However, caution is required when increasing the number of models or input modalities. There is a risk of overfitting, so careful consideration of appropriate model design (types and quantity of models) is necessary.

## 5. Conclusions

This study demonstrated that the combined sensors exhibited higher diagnostic efficiency compared to the individual modal sensors. Furthermore, the effectiveness of different sensors for detection varied depending on the specific disease, underscoring the synergistic effects of combining sensors. Additionally, this research underscores the potential of multimodal artificial intelligence.

## Figures and Tables

**Table 1:** Basic characteristics of the participants.

	Training/Development/Test Cases: N = 1051	Training/Development Cases: N = 839
Age	67.0 ± 15.7	67.2 ± 15.6
Male	574 (54.6%)	453 (54.0%)
Hypertension	688 (65.5%)	548 (65.3%)
Diabetes Mellitus	217 (20.7%)	168 (20.0%)
Dyslipidemia	351 (33.4%)	280 (33.4%)
Atrial Fibrillation at Enrollment	182 (17.3%)	146 (17.4%)
Heart Rate (Beats per Minute)	72.6 ± 14.1	72.6 ± 14.4
History of Stroke/TIA	135 (12.8%)	103 (12.3%)
History of Myocardial Infarction	124 (12.8%)	101 (12.0%)
History of CABG	59 (5.6%)	40 (4.8%)
LVEF (%)	58.7 ± 10.4	58.7 ± 10.4
Aortic Stenosis (no/I/II/III)	785/68/66/132 (74.7%/6.5%/6.3%/12.6%)	626/53/54/106 (74.6%/6.3%/6.4%/12.6%)
Aortic Regurgitation (no/I/II/III)	729/247/72/3 (69.4%/23.5%/6.9%/0.3%)	581/198/57/3 (69.3%/23.6%/6.8%/0.4%)
Mitral Stenosis (no/I/II/III)	968/67/15/1 (92.1%/6.4%/1.4%/0.1%)	778/48/12/1 (92.7%/5.7%/1.4%/0.1%)
Mitral Regurgitation (no/I/II/III)	469/385/130/67 (44.6%/36.6%/12.4%/6.4%)	376/306/104/53 (44.8%/36.5%/12.4%/6.3%)
Tricuspidal Stenosis (no/I/II/III)	1044/5/1/1 (99.3%/0.5%/0.1%/0.1%)	832/5/1/1 (99.2%/0.6%/0.1%/0.1%)
Tricuspidal Regurgitation (no/I/II/III)	463/456/103/29 (44.1%/43.4%/9.8%/2.8%)	382/346/85/26 (45.5%/41.2%/10.1%/3.1%)
PQ Interval (ms)	169 ± 36	169 ± 36
QRS Duration (ms)	100 ± 23	100 ± 24
QT Interval (ms)	403 ± 37	404 ± 38
QRS Axis (Degree)	31.8 ± 50.3	31.6 ± 49.7

CABG, coronary artery bypass grafting; LVEF, left ventricular ejection fraction; TIA, transient ischemic attack.

**Table 2:** Characteristics of the clinical validation participants.

	Clinical Validation Cases: N = 103
Age	73.1 ± 12.8
Male	46 (45.7%)
Hypertension	81 (78.6%)
Diabetes Mellitus	25 (24.3%)
Dyslipidemia	51 (49.5%)
Atrial Fibrillation at Enrollment	38 (36.9%)
Heart Rate (Beats per Minute)	76.0 ± 15.6
History of Stroke/TIA	8 (7.8%)
History of Myocardial Infarction	11 (10.7%)
History of CABG	14 (13.6%)
LVEF (%)	53.2 ± 12.4
Aortic Stenosis (no/I/II/III)	55/12/13/23 (53.4%/11.7%/12.6%/22.3%)
Aortic Regurgitation (no/I/II/III)	56/36/10/1 (54.4%/35.0%/9.7%/0.9%)
Mitral Stenosis (no/I/II/III)	83/15/5/0 (80.6%/14.6%/4.8%/0%)
Mitral Regurgitation (no/I/II/III)	23/39/23/18 (22.3%/37.9%/22.3%/17.5%)
Tricuspidal Stenosis (no/I/II/III)	103/0/0/0 (100%/0%/0%/0%)
Tricuspidal Regurgitation (no/I/II/III)	34/58/6/5 (33.0%/56.3%/5.8%/4.9%)
PQ Interval (ms)	182 ± 40
QRS Duration (ms)	104 ± 27
QT Interval (ms)	429 ± 44
QRS Axis (Degree)	15.2 ± 52.8

CABG, coronary artery bypass grafting; LVEF, left ventricular ejection fraction; TIA, transient ischemic attack.

**Table 3:** Data augmentation of ECG data.

Severity	Multiplying Factor
No AS	×3
Mild AS	
Moderate AS	
Severe AS	×20
No MR	×3
Mild MR	
Moderate MR	
Severe MR	×44
Normal LVEF	×3
Mildly reduced LVEF	
Moderately reduced LVEF	
Severely reduced LVEF	×42

**Table 4:** Stacking pattern for models.

Model	PCG			ECG		
	2RSB	Erb	Apex	12 Lead	Limb Lead	Precordial Lead
PCG3ch + ECG2pt	◯	◯	◯	-	◯	◯
PCG3ch + ECG1pt	◯	◯	◯	◯	-	-
PCG3ch	◯	◯	◯	-	-	-
ECG2pt	-	-	-	-	◯	◯

ECG, electrocardiogram; PCG, phonocardiogram; 2RSB, second right sternal border; ◯, utilized; -, not utilized.

**Table 5:** Results of bootstrapping in test cohort of aortic stenosis.

	AS			
Models	AUC	F1	Sensitivity	Specificity
ALLECG	0.780 ± 0.048	0.422 ± 0.066	0.766 ± 0.084	0.742 ± 0.033
LCECG	0.699 ± 0.060	0.402 ± 0.069	0.655 ± 0.095	0.779 ± 0.031
PCECG	0.800 ± 0.044	0.411 ± 0.073	0.573 ± 0.096	0.833 ± 0.028
PCG1 (2RSB)	0.974 ± 0.011	0.759 ± 0.060	0.923 ± 0.054	0.930 ± 0.018
PCG2 (Erb)	0.947 ± 0.016	0.625 ± 0.072	0.848 ± 0.074	0.881 ± 0.024
PCG3 (Apex)	0.909 ± 0.031	0.568 ± 0.070	0.847 ± 0.071	0.844 ± 0.026
RF(ECG2pt)	0.794 ± 0.047	0.423 ± 0.074	0.574 ± 0.096	0.844 ± 0.027
RF(PCG3ch)	0.971 ± 0.010	0.766 ± 0.059	0.961 ± 0.039	0.925 ± 0.019
RF(PCG3ch + ECG1pt)	0.970 ± 0.010	0.790 ± 0.057	0.961 ± 0.039	0.935 ± 0.018
RF(PCG3ch + ECG2pt)	0.971 ± 0.010	0.804 ± 0.056	0.961 ± 0.039	0.941 ± 0.017
XGB(ECG2pt)	0.756 ± 0.052	0.429 ± 0.075	0.574 ± 0.096	0.849 ± 0.027
XGB(PCG3ch)	0.969 ± 0.015	0.778 ± 0.058	0.961 ± 0.039	0.930 ± 0.019
XGB(PCG3ch + ECG1pt)	0.974 ± 0.010	0.767 ± 0.060	0.961 ± 0.039	0.925 ± 0.019
XGB(PCG3ch + ECG2pt)	0.964 ± 0.015	0.754 ± 0.060	0.961 ± 0.039	0.919 ± 0.020

ALLECG, 12-lead ECG; LCECG, limb-lead ECG; PCECG, precordial-lead ECG; RF, Random Forest; XGB, XGBoost; ECG, electrocardiogram; PCG, phonocardiogram. For ECG1pt, ECG2pt, and PCG3ch, see Figure 4.

**Table 6:** Results of bootstrapping in test cohort of mitral regurgitation.

	MR			
Models	AUC	F1	Sensitivity	Specificity
ALLECG	0.678 ± 0.079	0.214 ± 0.081	0.361 ± 0.136	0.864 ± 0.025
LCECG	0.717 ± 0.057	0.207 ± 0.059	0.645 ± 0.131	0.683 ± 0.033
PCECG	0.787 ± 0.060	0.279 ± 0.067	0.787 ± 0.116	0.732 ± 0.032
PCG1(2RSB)	0.597 ± 0.063	0.168 ± 0.044	0.787 ± 0.114	0.470 ± 0.035
PCG2 (Erb)	0.714 ± 0.080	0.225 ± 0.074	0.497 ± 0.142	0.797 ± 0.029
PCG3 (Apex)	0.629 ± 0.082	0.181 ± 0.061	0.500 ± 0.142	0.722 ± 0.032
RF(ECG2pt)	0.695 ± 0.053	0.208 ± 0.058	0.645 ± 0.131	0.683 ± 0.033
RF(PCG3ch)	0.699 ± 0.071	0.204 ± 0.059	0.641 ± 0.136	0.677 ± 0.033
RF(PCG3ch + ECG1pt)	0.722 ± 0.071	0.241 ± 0.063	0.716 ± 0.125	0.707 ± 0.033
RF(PCG3ch + ECG2pt)	0.736 ± 0.067	0.191 ± 0.056	0.643 ± 0.133	0.647 ± 0.034
XGB(ECG2pt)	0.652 ± 0.071	0.193 ± 0.057	0.576 ± 0.138	0.697 ± 0.032
XGB(PCG3ch)	0.685 ± 0.078	0.199 ± 0.058	0.641 ± 0.136	0.667 ± 0.033
XGB(PCG3ch + ECG1pt)	0.729 ± 0.072	0.227 ± 0.064	0.641 ± 0.136	0.722 ± 0.032
XGB(PCG3ch + ECG2pt)	0.757 ± 0.063	0.242 ± 0.068	0.641 ± 0.136	0.747 ± 0.030

For abbreviations, see Table 5.

**Table 7:** Results of bootstrapping in test cohort of LVEF.

	LVEF			
Models	AUC	F1	Sensitivity	Specificity
ALLECG	0.904 ± 0.035	0.385 ± 0.081	0.857 ± 0.100	0.818 ± 0.028
LCECG	0.842 ± 0.045	0.284 ± 0.066	0.857 ± 0.100	0.707 ± 0.033
PCECG	0.896 ± 0.033	0.451 ± 0.087	0.862 ± 0.097	0.863 ± 0.025
PCG1	0.775 ± 0.065	0.175 ± 0.109	0.142 ± 0.095	0.970 ± 0.012
PCG2	0.712 ± 0.069	0.196 ± 0.054	0.709 ± 0.127	0.611 ± 0.034
PCG3	0.824 ± 0.052	0.229 ± 0.069	0.568 ± 0.141	0.762 ± 0.030
RF(ECG2pt)	0.904 ± 0.023	0.340 ± 0.069	1.000 ± 0.000	0.727 ± 0.032
RF(PCG3ch)	0.775 ± 0.057	0.246 ± 0.062	0.783 ± 0.118	0.677 ± 0.033
RF(PCG3ch + ECG1pt)	0.895 ± 0.043	0.391 ± 0.081	0.857 ± 0.100	0.823 ± 0.027
RF(PCG3ch + ECG2pt)	0.881 ± 0.044	0.433 ± 0.085	0.857 ± 0.100	0.853 ± 0.024
XGB(ECG2pt)	0.925 ± 0.023	0.365 ± 0.075	0.931 ± 0.074	0.778 ± 0.030
XGB(PCG3ch)	0.741 ± 0.070	0.203 ± 0.056	0.711 ± 0.128	0.627 ± 0.035
XGB(PCG3ch + ECG1pt)	0.894 ± 0.040	0.436 ± 0.091	0.785 ± 0.118	0.874 ± 0.023
XGB(PCG3ch + ECG2pt)	0.906 ± 0.033	0.436 ± 0.089	0.787 ± 0.115	0.873 ± 0.023

For abbreviations, see Table 5.

## Data Availability

The patient data underlying this article cannot be shared publicly to protect the privacy of the individuals who participated in the study. As a corporate policy of Mitsubishi Electric, we generally do not disclose source codes. However, if we are approached with legitimate grounds and a clear purpose, we are open to considering such requests.

## Supplementary Materials

The following supporting information can be downloaded at: https://www.mdpi.com/article/10.3390/s23249834/s1, Table S1: Training conditions of CNN.
