# Supplementary material for: Improving Valvular Pathologies and Ventricular Dysfunction Diagnostic Efficiency Using Combined Auscultation and Electrocardiography Data: A Multimodal AI Approach
Source: Sensors (Basel). 2023 Dec 14;23(24):9834. doi: 10.3390/s23249834 (PMC10748155; doi:10.3390/s23249834)
Supplement: Supplementary file 1 [file sensors-23-09834-s001.zip › sensors-2719564-supplementary.pdf]

Improving Valvular Pathologies and Ventricular Dysfunction Diagnostic Efficiency Using Combined Auscultation and Electrocardiography Data: A Multimodal AI Approach

Supplementary information

Supplementary Table 1. Training conditions of CNN

**Equipment**	**CPU**	**Intel(R) Xeon(R) CPU E5-1650 v2 @ 3.50GHz**
	**GPU**	GeForce GTX 1080 Ti
**Learning condition**	**Epoch number**	100
	**Learning rate**	0.001
	**Optimizer**	Adam
